# The Challenge of Determining the Etiology of Encephalopathy in an Elderly Patient

**DOI:** 10.1002/ccr3.73026

**Published:** 2026-06-25

**Authors:** Vlad Alexandru Ionescu, Gina Gheorghe, Roxana‐Manuela Vasile, Claudia‐Lucia Ionescu, Mihaela Bazac, Viorica Ileana Bumbea, Alina Valentina Dobrota, Ioana‐Alexandra Baban, Alexandru Barbu, Camelia Cristina Diaconu

**Affiliations:** ^1^ Faculty of Medicine University of Medicine and Pharmacy Carol Davila Bucharest Bucharest Romania; ^2^ Internal Medicine Department Clinical Emergency Hospital of Bucharest Bucharest Romania; ^3^ Department of Nephrology Clinical Emergency Hospital of Bucharest Bucharest Romania; ^4^ Academy of Romanian Scientists Bucharest Romania

**Keywords:** differential diagnosis, encephalopathy, hypercalcemia, malignancy, metabolic causes

## Abstract

Encephalopathy is a heterogeneous clinical syndrome with numerous neurological and systemic etiologies. We report the case of a 67‐year‐old man, a chronic ethanol consumer, admitted with a one‐week history of confusional syndrome. Initial laboratory tests revealed severe hypercalcemia, acute kidney injury, mild anemia, and hyperproteinemia. Serum ammonia levels were within normal limits, reducing the likelihood of hepatic encephalopathy despite the patient's chronic ethanol use. Cerebral imaging excluded acute cerebrovascular events, while abdominal ultrasound demonstrated splenomegaly and normal‐sized kidneys. Serum protein electrophoresis and immunofixation identified a monoclonal IgG‐kappa component, strongly suggestive of multiple myeloma. The patient's neurocognitive symptoms improved rapidly following systemic corticosteroid therapy and fluid–electrolyte rebalancing, confirming hypercalcemia as the primary mechanism of encephalopathy. Hypercalcemic encephalopathy as an initial manifestation of multiple myeloma is exceedingly rare. This case highlights the diagnostic complexity in elderly patients with multiple potential contributors to altered mental status, including chronic ethanol exposure and renal dysfunction. The presence of normal renal dimensions, unexplained hypercalcemia, and a monoclonal component should prompt evaluation for plasma cell malignancy. Early recognition of hypercalcemia‐induced encephalopathy and prompt investigation for underlying hematologic malignancy are essential to prevent irreversible organ damage and to ensure timely initiation of specific therapy.

## Key Clinical Message

Hypercalcemia‐induced encephalopathy may be the initial presentation of multiple myeloma in elderly patients, and the combination of unexplained hypercalcemia, acute kidney injury, and a monoclonal component should prompt early evaluation for plasma cell malignancy.

## 1 Introduction

Encephalopathy is a clinical syndrome characterized by an altered mental status, with impairment of cognition and level of alertness [1]. Its underlying causes are diverse, and the differential diagnosis can be guided by a thorough medical history—paying close attention to the pace of onset, symptom progression, and total duration—along with a detailed physical examination and appropriate laboratory or imaging investigations [1, 2]. As patients with encephalopathy frequently cannot provide reliable information regarding their condition, collateral history obtained from family members or other close contacts is essential [1, 2].

According to the temporal evolution of symptoms, encephalopathy can be classified into four major categories:

- Hyperacute—symptoms developing within seconds to minutes.
- Acute—symptoms evolving over several hours to a few days.
- Subacute—symptoms progressing over several weeks to months.
- Chronic—symptoms with a slowly progressive course over months or years [1, 2].

Encephalopathy may result from primary neurological disorders as well as systemic, toxic, or metabolic conditions [1, 2]. Neurological causes include cerebrovascular disease, seizures, meningoencephalitis, primary or metastatic brain tumors, multiple sclerosis, and neurodegenerative disorders [1]. Systemic causes include sepsis, acute azotemia, electrolyte or acid–base disturbances, hyperammonemia, hyperglycemia, hypoglycemia, thyroid dysfunction, acute vitamin B deficiency, hypertensive crisis, and medication‐induced toxicity [2, 3]. Hepatic encephalopathy is a frequent cause of metabolic encephalopathy. Ammonia and inflammation are the major contributors to its pathogenesis, and in patients with liver cirrhosis, the underlying mechanisms involve low‐grade cerebral edema, oxidative and nitrosative stress, and disturbances of oscillatory brain networks [4, 5].

Altered mental status in patients with multiple myeloma may occur secondary to several mechanisms, including hypercalcemia, uremia, hyperviscosity, hyperammonemia, or leptomeningeal myelomatosis [5, 6, 7]. Hypercalcemia represents an important and potentially reversible cause of metabolic encephalopathy. Other common etiologies of hypercalcemia include solid malignancies, medications such as thiazide diuretics, lithium or tamoxifen, granulomatous diseases, and the milk–alkali syndrome [8, 9, 10].

In multiple myeloma, the predominant cause of hypercalcemia is tumor‐induced bone destruction through increased osteoclastic resorption [11]. This process is mediated by cytokines strongly expressed or secreted by myeloma cells or by other cells within the bone marrow microenvironment, such as tumor necrosis factor (TNF), receptor activator of nuclear factor‐κB ligand (RANKL), and macrophage inflammatory protein‐1α (MIP‐1α) [11]. An additional mechanism contributing to hypercalcemia in these patients is irreversible renal impairment with increased tubular calcium reabsorption [11]. A major difference between hypercalcemia associated with multiple myeloma and hypercalcemia of solid malignancies is that, in myeloma, serum calcium elevation is largely independent of parathyroid hormone–related protein (PTHrP) levels, whereas hypercalcemia in solid tumors is almost always driven by excessive PTHrP secretion [11]. Consequently, myeloma‐associated hypercalcemia typically responds rapidly to corticosteroid therapy due to prompt suppression of myeloma cell proliferation, unlike hypercalcemia in other malignancies [11].

The pathogenesis of hypercalcemia‐induced encephalopathy is not fully understood, but it is thought to involve alterations in neurotransmission, endothelial dysfunction, cerebral vasospasm, and the development of cerebral edema, all contributing to mental status changes [11, 12].

Within this complex pathophysiological context, we present the case of an elderly patient in whom encephalopathy represented the initial manifestation of previously undiagnosed multiple myeloma, occurring in the setting of severe hypercalcemia and acute kidney injury (AKI).

## 2 Case History/Examination

A 67‐year‐old patient, chronic ethanol consumer and former smoker (20 pack‐years), abstinent for approximately 20 years, with a history of apical myocardial infarction and left occipital ischemic stroke (2017), grade II arterial hypertension, type 2 diabetes mellitus under oral antidiabetic therapy, chronic venous insufficiency of the lower limbs, and previous cholecystectomy (2014), was admitted for a confusional syndrome persisting for about one week.

Clinical examination revealed a patient in fair general condition, conscious, poorly cooperative, temporo‐spatially disoriented, with no focal neurological signs, no neck stiffness, afebrile, obese (BMI 33.3 kg/m^2^), normally colored but slightly dehydrated skin and mucosae, an atrophic scar in the right hypochondrium (post‐cholecystectomy), hypotonic and hypokinetic muscular system, musculoskeletal system morpho‐functionally preserved, present vesicular murmur bilaterally without rales, SpO_2_ 96% on room air, rhythmic heart sounds without murmurs, blood pressure 150/78 mmHg, ventricular rate 75/min, no signs of peripheral congestion, abdomen mobile with respiration, non‐tender spontaneously or on palpation, preserved bowel movements, normal stool, negative Giordano's sign bilaterally, preserved diuresis with normal micturition.

## 3 Methods

Laboratory investigations showed mild normochromic, normocytic anemia (hemoglobin 10.8 g/dL, MCV 86.60 fL, MCH 29.10 pg., MCHC 33.60 g/dL), mild thrombocytopenia (platelets 131,000/μL), inflammatory syndrome (ESR 92 mm/h, CRP 6.38 mg/L), azotemia (creatinine 3.55 mg/dL, urea 124.8 mg/dL), hypercalcemia (ionic calcium 2.62 mmol/L, total calcium 16.69 mg/dL), hyperproteinemia (total protein 8.7 g/dL), hyperuricemia (uric acid 13.47 mg/dL), hyperphosphatemia (phosphorus 5.04 mg/dL), LDH 166 U/L, and hypocholesterolemia (total cholesterol 70 mg/dL). Venous blood gas analysis confirmed hypercalcemia, without other significant alterations (pH 7.43, HCO₃^−^ 29.9 mmol/L, anion gap 6 mmol/L, base excess 5.2 mmol/L, lactate 0.6 mmol/L). Considering the neurological symptoms and the patient's cerebrovascular history, a cerebral CT scan was performed and excluded acute lesions.

Electrocardiography revealed sinus rhythm, ventricular rate 75/min, normal PR interval, narrow fragmented QRS in V1–V4, DIII, aVF, with qS morphology in V1–V4 and negative T waves in V1–V4. Transthoracic echocardiography showed a left ventricle with dimensions at the upper limit of normal, mildly concentrically hypertrophied, with severe systolic dysfunction (LVEF 35%), hypokinesia of the posterior interventricular septum (apical third), apical wall (two apical thirds), anterior wall (entire length), and inferior wall (apical third), no hemodynamically significant valvular disease, right ventricle not dilated with preserved longitudinal systolic function, and no pericardial effusion. Serial cardiac enzyme testing revealed no changes, while NT‐proBNP was markedly elevated at 9870 pg/mL.

**FIGURE 1:**
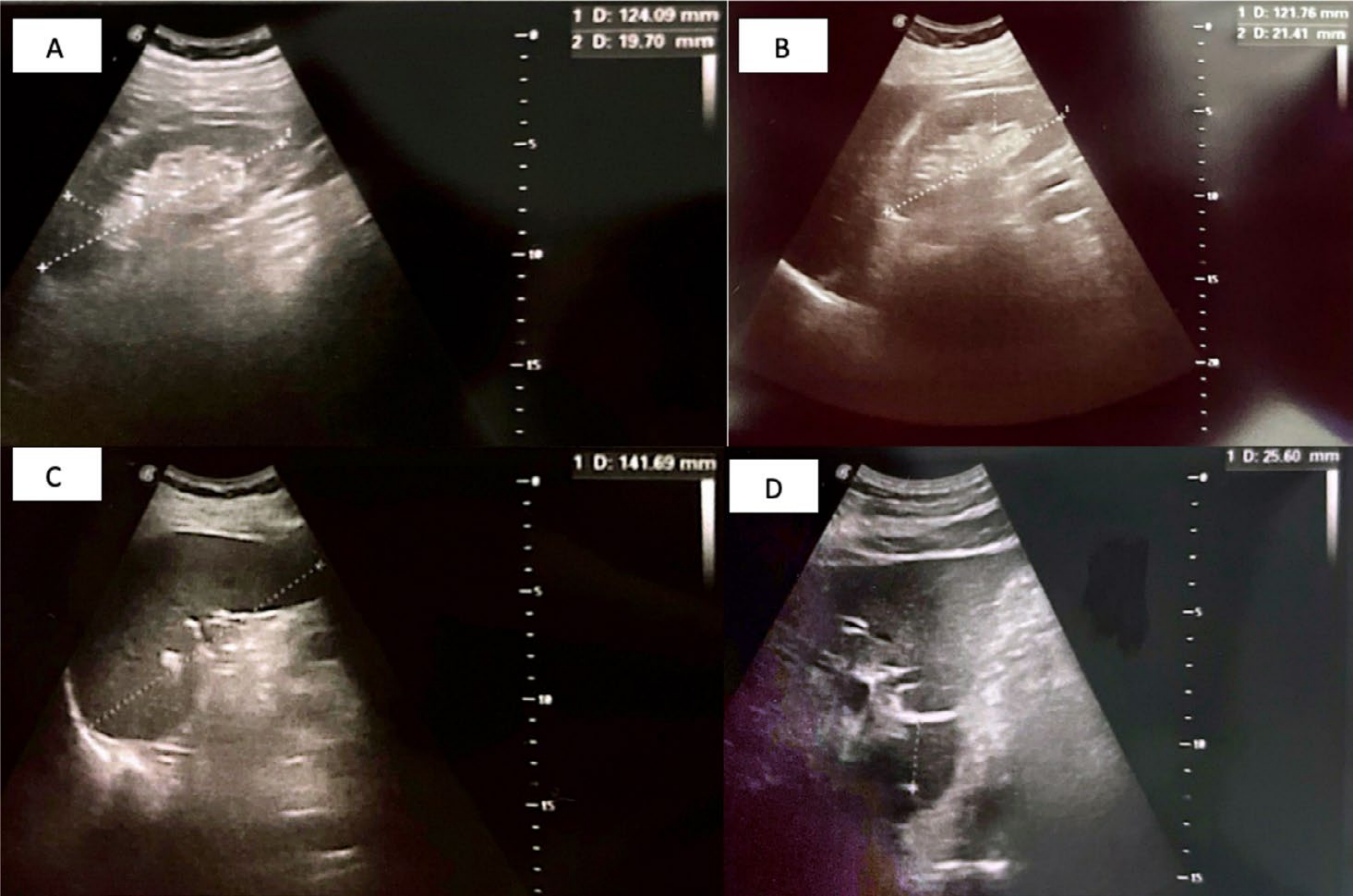
Abdominal ultrasound. (A) Right kidney with a longitudinal axis of 12.4 cm and parenchymal thickness of 2.0 cm, without renal calculi or pelvicalyceal dilatation. (B) Left kidney with a longitudinal axis of 12.2 cm and parenchymal thickness of 2.1 cm, likewise without calculi or pelvicalyceal dilatation. (C) Spleen measuring 14.1 cm in maximal diameter, with homogeneous echotexture. (D) Left hepatic lobe (anteroposterior diameter ≈ 6.0 cm) and caudate lobe (diameter ≈ 2.6 cm), both showing homogeneous echotexture.

Abdominopelvic ultrasound revealed hepatomegaly (left lobe 6 cm, caudate lobe 2.6 cm, right lobe prerenal diameter 18 cm), homogeneous structure with diffusely increased echogenicity, slightly irregular contour, portal vein diameter 14 mm with preserved flow on Doppler examination, non‐dilated extra‐ and intrahepatic bile ducts, pancreas of normal size with fatty infiltration, splenomegaly (anteroposterior diameter 14.1 cm) with normal echogenicity, kidneys of normal dimensions with microcalculi, without pelvicalyceal dilatation (Figure 1).

Urinary studies: urinalysis without pathological findings; urinary sediment with rare squamous epithelial cells, rare leukocytes, no flora; urine culture negative. Biochemistry of 24‐h urine showed moderate proteinuria (0.7 g/24 h) without other abnormalities.

To evaluate the etiology of mild anemia, upper gastrointestinal endoscopy showed antral gastritis without bleeding‐risk lesions. Peripheral blood smear revealed no significant abnormalities (neutrophils 61%, eosinophils 1%, lymphocytes 30%, monocytes 8%), platelets isolated, frequent in small clusters, anisocytosis with macrotrombocytes, normocytic normochromic erythrocytes.

Following multidisciplinary evaluation (internal medicine, neurology, nephrology, cardiology, hematology), the following provisional diagnoses were established: hypercalcemic encephalopathy, suspicion of multiple myeloma, AKI, hepatosplenomegaly of uncertain etiology, heart failure with reduced ejection fraction, history of apical myocardial infarction and left occipital ischemic stroke (2017), grade II arterial hypertension, type 2 diabetes mellitus under oral therapy, chronic venous insufficiency of the lower limbs, and history of cholecystectomy (2014).

Therapy was initiated with fluid and electrolyte rebalancing, loop diuretic, and systemic corticosteroid, leading to progressive normalization of serum calcium values and remission of encephalopathy. The patient also received antiplatelet therapy, statin, beta‐blocker, calcium channel blocker, and proton pump inhibitor, with favorable evolution.

**FIGURE 2:**
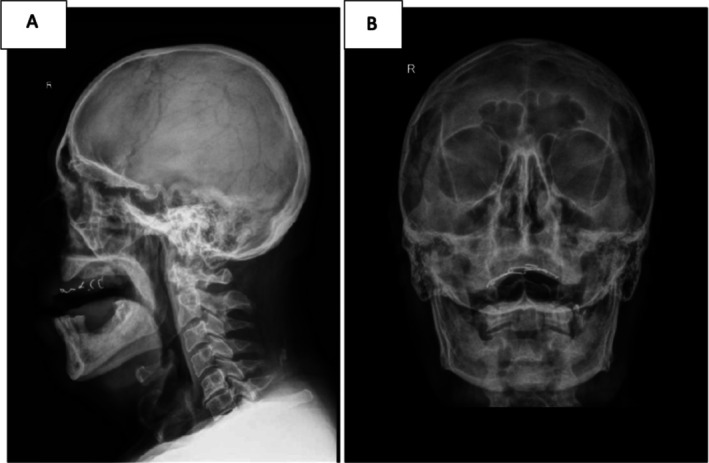
Lateral (A) and anteroposterior (B) skull radiographs showing no osteolytic lesions, no focal bone defects, and preserved calvarial architecture. The cortical and trabecular bone structures appear intact, with no evidence of punched‐out lesions typically associated with multiple myeloma.

To establish a definitive diagnosis, further investigations were performed: skull radiography (no significant findings—Figure 2), serum protein electrophoresis and immunoelectrophoresis, which revealed hyperproteinemia, hypoalbuminemia, decreased A/G ratio, monoclonal component in the beta‐2 region and decreased gamma globulin fraction (Table 1). Immunoelectrophoresis demonstrated a monoclonal IgG‐kappa immunoglobulin (Table 1).

**TABLE 1:** Immunoelectrophoresis and serum protein electrophoresis findings.

Parameter	Result		
Immunoglobulin IgA	Absence of a monoclonal band		
Immunoglobulin IgM	Absence of a monoclonal band		
Immunoglobulin IgG	Presence of a monoclonal band		
Kappa light chains	Presence of a monoclonal band		
Lambda light chains	Absence of a monoclonal band		
**Serum proteins**	**Serum value**	**Reference range**	**Deviation from normal range**
Albumin/Globulin ratio	0.62	1.0–1.5	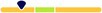
Total proteins	8.8 g/dl	5.7–8.2	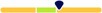
Albumin	3.38 g/dL (38.4%)	55.8%–66.1%	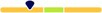
Alpha1‐globulins	0.34 g/dL (3.9%)	2.9%–4.9%	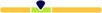
Alpha2‐globulins	0.66 g/dL (7.5%)	7.1%–11.8%	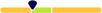
Beta1‐globulins	0.28 g/dL (3.2%)	4.7%–7.2%	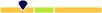
Beta2‐globulins	3.74 g/dL (42.5%)	3.2%–6.5%	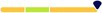
Gamma‐globulins	0.4 g/dl (4.5%)	11.1%–18.8%	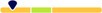

*Note:* Deviation from normal range is illustrated graphically: bars extending to the left or right of the reference interval indicate decreased or increased values, respectively.

All these investigations were highly suggestive of multiple myeloma, and at this stage the patient fulfilled several CRAB (C—hypercalcemia, R—renal failure, A—anemia, B—bone lesions) criteria—specifically hypercalcemia, renal impairment, and mild anemia—raising strong suspicion for symptomatic disease. Consequently, he was transferred to a hematology department for completion of the diagnostic workup and initiation of appropriate, disease‐specific therapy.

## 4 Conclusions and Results

In conclusion, this case highlights the diagnostic and therapeutic complexity of multiple myeloma in an elderly patient with multiple comorbidities, particularly in the context of an atypical clinical presentation dominated by hypercalcemic encephalopathy and AKI. We considered hypercalcemia to be the primary mechanism underlying the encephalopathy, given the prompt improvement following systemic corticosteroid therapy and aggressive fluid and electrolyte rebalancing, despite the absence of significant improvement in azotemic parameters. The subsequent identification of a monoclonal IgG‐kappa component and its correlation with the clinical picture facilitated rapid orientation toward the correct diagnosis.

A key element emphasized by this case is that the renal dysfunction was an early manifestation of previously unrecognized multiple myeloma, as suggested by the normal renal dimensions and absence of ultrasonographic features consistent with advanced chronic nephropathy. This underscores the importance of maintaining a high index of suspicion for multiple myeloma in elderly patients presenting with recent‐onset renal impairment unexplained by structural pathology, particularly in the presence of hypercalcemia and mild proteinuria.

Severe hypercalcemia was the main determinant of the neurological and renal manifestations in this case, and its management was significantly complicated by coexisting heart failure with reduced ejection fraction. This necessitated a carefully balanced therapeutic approach, in which intravenous hydration—essential for the treatment of hypercalcemia—required adjustment to avoid volume overload. The patient's favorable evolution demonstrates the value of multidisciplinary collaboration among internal medicine, nephrology, cardiology, neurology, and hematology.

The present case reinforces the need for rigorous application of the International Myeloma Working Group (IMWG) criteria when evaluating patients with suspected multiple myeloma and highlights the central role of modern diagnostic tools—serum and urine electrophoresis with immunofixation, serum free light chain measurement, and whole‐body imaging—in the early identification of disease manifestations. Early diagnosis is crucial, as prompt initiation of targeted therapy may prevent progression to irreversible organ damage, improve patient outcomes, and reduce mortality.

## 5 Discussion

Multiple myeloma is a neoplasm characterized by the uncontrolled monoclonal proliferation of plasma cells in the bone marrow [13]. This condition is defined by excessive production of monoclonal immunoglobulins, which leads to secondary suppression of normal immunoglobulin synthesis [13]. Recent epidemiological data have reported a progressive annual increase in the number of multiple myeloma cases, with this malignancy accounting for approximately 0.9% of all cancer diagnoses worldwide [14]. According to the most recent Global Cancer Observatory report, an estimated 190,000 new cases of multiple myeloma and 122,000 associated deaths occur annually [14]. The incidence shows geographic variability, with 39.3% of cases reported in Asia, 26.7% in Europe, 19.7% in Northern America, 8.1% in Latin America and the Caribbean, 4.8% in Africa, and 1.5% in Oceania [15]. The disease is approximately 1.5 times more frequent in men [16], with a cumulative risk of being diagnosed with multiple myeloma by the age of 74 years of 0.24% in men and 0.17% in women [16]. The median age at diagnosis is 69 years, more than 60% of cases being diagnosed in patients older than 65 years and fewer than 15% in those younger than 55 years [16, 17]. Our patient fits this epidemiological profile.

Patients with multiple myeloma frequently present with CRAB features [18]. At the time of diagnosis, 79% of patients present with osteolytic lesions, 73% with anemia, and 19% with AKI [19]. Initial presentation with hypercalcemic encephalopathy in a patient with multiple myeloma is exceedingly rare, with only a few cases reported in the literature [7, 20, 21, 22], which represents one of the distinctive aspects of our case. In addition, the recent history of ischemic stroke slightly complicated the diagnostic process, as the confusional state was initially interpreted in the context of a cerebrovascular event and dehydration.

In the setting of multiple potential contributors to the confusional syndrome (cerebrovascular disease, uremia, hypercalcemia, chronic ethanol use), we considered hypercalcemia to be the main mechanism, based on the rapid improvement of neuropsychiatric symptoms after initiation of systemic corticosteroid therapy and more aggressive fluid and electrolyte rebalancing, in the absence of a significant improvement in renal function. This underscores the importance of investigating multiple myeloma in elderly patients with newly developed renal dysfunction, hypercalcemia, mild proteinuria, and normal‐sized kidneys.

The initial laboratory evaluation in a patient with suspected multiple myeloma should include a complete blood count with differential, serum calcium, serum creatinine, lactate dehydrogenase, serum free light chains, and β2‐microglobulin levels [19]. Serum and 24‐h urine protein electrophoresis with immunofixation are also mandatory [19, 23]. Measurement of serum free light chains enables quantification of kappa and lambda light chain levels that may contribute to organ damage [24]. In approximately 86% of multiple myeloma cases, serum protein electrophoresis identifies a monoclonal protein (an atypical immunoglobulin) [25]. Determining monoclonal protein and serum free light chain levels at diagnosis is essential for assessing disease burden and subsequent response to therapy [23].

Imaging assessment plays a central role both in diagnosis and in staging. Current guidelines recommend whole‐body imaging using low‐dose computed tomography (LD‐CT), positron emission tomography–computed tomography (PET‐CT), or magnetic resonance imaging (MRI) [23]. These modalities are clearly superior to conventional skeletal survey for the detection of lytic lesions, vertebral compression fractures, or pathological fractures. In resource‐limited settings, conventional skeletal radiography remains an acceptable alternative [23]. In our case, skull radiographs and cranial CT did not demonstrate osteolytic lesions; however, this does not exclude myeloma bone disease and underlines the need for completion of whole‐body imaging.

Diagnostic confirmation requires bone marrow aspiration and biopsy, with assessment of plasma cell morphology and quantification of CD138+ plasma cells by immunohistochemistry, flow cytometry, fluorescence in situ hybridization (FISH), and conventional cytogenetics [23, 26]. The diagnosis of multiple myeloma is based on the presence of ≥ 10% clonal plasma cells in the bone marrow or a biopsy‐proven plasmacytoma, together with one or more myeloma‐defining events [17, 18]. These include the presence of one or more CRAB features or one or more biomarkers of malignancy [27, 28]. The three biomarkers included in the current definition of multiple myeloma are: an involved/uninvolved serum free light chain (FLC) ratio ≥ 100, provided the involved FLC is ≥ 100 mg/L; ≥ 60% clonal plasma cells in the bone marrow; or more than one focal lesion on MRI [27, 28]. In our patient, the association of a clearly defined monoclonal IgG‐kappa component with severe hypercalcemia, AKI, and mild anemia fulfilled the IMWG criteria for symptomatic multiple myeloma, thereby justifying urgent transfer to a hematology center for histologic confirmation, risk stratification (ISS/R‐ISS), and initiation of specific therapy.

Another particular feature of this case was the management of severe hypercalcemia in a patient with heart failure with reduced ejection fraction (LVEF 35%) and renal impairment. Aggressive intravenous hydration, which is a cornerstone of hypercalcemia treatment, carries a significant risk of volume overload in this context, necessitating close hemodynamic monitoring and individualized titration of loop diuretics. The favorable clinical evolution, with resolution of encephalopathy and normalization of serum calcium, underscores the effectiveness of a multidisciplinary approach.

## Author Contributions

**Vlad Alexandru Ionescu:** conceptualization, investigation, methodology, resources, software, writing – original draft, writing – review and editing. **Gina Gheorghe:** conceptualization, investigation, methodology, resources, software, writing – original draft, writing – review and editing. **Roxana‐Manuela Vasile:** investigation, resources. **Claudia‐Lucia Ionescu:** investigation, resources. **Mihaela Bazac:** investigation, resources. **Viorica Ileana Bumbea:** investigation, resources. **Alina Valentina Dobrota:** investigation, resources. **Ioana‐Alexandra Baban:** investigation, methodology, resources, software. **Alexandru Barbu:** investigation, methodology, resources, software. **Camelia Cristina Diaconu:** resources, supervision, visualization, writing – review and editing.

## Funding

The authors have nothing to report.

## Ethics Statement

As a single‐case report with the patient's signed consent, no other ethical review was required.

## Consent

Oral and written informed consent was obtained from the patient for the publication of clinical data and images related to the disease in this manuscript. The identity of the patient has been anonymized to protect the privacy of the patient.

## Conflicts of Interest

The authors declare no conflicts of interest.

## Data Availability

Data are contained within the article.

## References

[1] M. G. Erkkinen and A. L. Berkowitz , “A Clinical Approach to Diagnosing Encephalopathy,” American Journal of Medicine 132 (2019): 1142–2247, 10.1016/j.amjmed.2019.07.001. 31330129

[2] E. F. M. Wijdicks , “Identifying Encephalopathies From Acute Metabolic Derangements,” Journal of Internal Medicine 292 (2022): 846–857, 10.1111/joim.13538. 35809045

[3] L. L. Guennec , C. Marois , S. Demeret , E. F. M. Wijdicks , and N. Weiss , “Toxic‐Metabolic Encephalopathy in Adults: Critical Discussion and Pragmatical Diagnostic Approach,” Revue Neurologique 178 (2022): 93–104, 10.1016/j.neurol.2021.11.007. 34996631

[4] D. Haussinger , R. K. Dhiman , V. Felipo , et al., “Hepatic Encephalopathy,” Nature Reviews Disease Primers 8 (2022): 43, 10.1038/s41572-022-00366-6. 35739133

[5] G. Gheorghe , C. C. Diaconu , S. Bungau , N. Bacalbasa , N. Motas , and V. A. Ionescu , “Biliary and Vascular Complications After Liver Transplantation‐From Diagnosis to Treatment,” Medicina 59 (2023): 850, 10.3390/medicina59050850. 37241082 PMC10221850

[6] S. K. Sharma , D. Choudhary , A. Handoo , et al., “An Unusual Cause of Anemia and Encephalopathy,” Mediterranean Journal of Hematology and Infectious Diseases 7 (2015): e2015036, 10.4084/MJHID.2015.036. 25960864 PMC4418384

[7] D. S. Meena , G. K. Bohra , M. K. Garg , A. Purohit , D. Kumar , and S. Tripathi , “Hypercalcemic Encephalopathy as an Initial Presentation of Multiple Myeloma,” Case Reports in Emergency Medicine 2020 (2020): 4746865, 10.1155/2020/4746865. 32089905 PMC7031711

[8] M. D. Walker and E. Shane , “Hypercalcemia: A Review,” Jama 328 (2022): 1624–1636, 10.1001/jama.2022.18331. 36282253

[9] P. Bartkiewicz , D. Kunachowicz , M. Filipski , A. Stebel , J. Ligoda , and N. Rembialkowska , “Hypercalcemia in Cancer: Causes, Effects, and Treatment Strategies,” Cells 13 (2024): 1051, 10.3390/cells13121051. 38920679 PMC11202131

[10] A. Ray , A. Kar , B. K. Ray , and S. Dubey , “Hypercalcaemic Encephalopathy as a Presenting Manifestation of Sarcoidosis,” BML Case Reports 14 (2021): e241246, 10.1136/bcr-2020-241246. PMC8454277 34544697

[11] B. O. Oyajobi , “Multiple Myeloma/Hypercalcemia,” Arthritis Research & Therapy 9 (2007): 1–6, 10.1186/ar2168. PMC1924519 17634143

[12] B. S. K. Prusty , K. K. Ramineni , G. K. M. Reddy , R. K. Jakkani , and U. B. Chakrahari , “Hypercalcemic Encephalopathy as the Presenting Manifestation of Sarcoidosis,” Indian Journal of Endocrinology and Metabolism 23 (2019): 384–385, 10.4103/ijem.IJEM_206_19. 31641647 PMC6683695

[13] D. Kazandjian , “Multiple Myeloma Epidemiology and Survival: A Unique Malignancy,” Seminars in Oncology 43 (2016): 676–681, 10.1053/j.seminoncol.2016.11.004. 28061985 PMC5283695

[14] Q. Hou , X. Li , H. Ma , D. Fu , and A. Liao , “A Systematic Epidemiological Trends Analysis Study in Global Burden of Multiple Myeloma and 29 Years Forecast,” Scientific Reports 15 (2024): 2204, 10.1038/s41598-024-83630-x. PMC11739580 39820043

[15] International Agency for Research on Cancer , accessed on 18 November 2025, https://gco.iarc.who.int/media/globocan/factsheets/cancers/35‐multiple‐myeloma‐fact‐sheet.pdf.

[16] S. A. Padala , A. Barsouk , A. Barsouk , et al., “Epidemiology, Staging, and Management of Multiple Myeloma,” Medical Sciences (Basel) 9 (2021): 3, 10.3390/medsci9010003. PMC7838784 33498356

[17] L. Zhue , X. Lin , Z. Fan , et al., “Global, Regional and National Epidemiological Trends of Multiple Myeloma From 1990 to 2021: A Systematic Analysis of the Global Burden of Disease Study 2021,” Frontiers in Public Health 13 (2025): 1527198, 10.3389/fpubh.2025.1527198. 39931304 PMC11807829

[18] S. V. Rajkumar and S. Kumar , “Multiple Myeloma: Diagnosis and Treatment,” Mayo Clinic Proceedings 91 (2016): 101–119, 10.1016/j.mayocp.2015.11.007. 26763514 PMC5223450

[19] A. J. Cowan , D. J. Green , M. Kwok , et al., “Diagnosis and Management of Multiple Myeloma: A Review,” Jama 327 (2022): 464–477, 10.1001/jama.2022.0003. 35103762

[20] C. M. Klomp , M. W. Van Den Broek , J. Buijs , and R. Beekman , “Reversible Posterior Leucoencephalopathy due to Hypercalcaemia,” Nederlands Tijdschrift Voor Geneeskunde 150 (2006): 505–508. 16553051

[21] A. Fayaz , A. H. Shah , M. A. Khan , and S. Parveen , “Multiple Myeloma Presenting as Hypercalcemia‐Induced Acute Severe Pancreatitis: A Case Report,” Gastro Hep Advances 4 (2024): 100549, 10.1016/j.gastha.2024.09.004. 40520417 PMC12166718

[22] G. Sandhu , A. A. Farias , A. Ranade , and I. Meisels , “Altered Mental Status in a Case of Multiple Myeloma Not Related to a Metabolic Cause,” Clinical Kidney Journal 2 (2009): 434–435, 10.1093/ndtplus/sfp083. PMC4421399 25949373

[23] S. V. Rajkumar , “Multiple Myeloma: Diagnosis and Treatment,” American Journal of Hematology 99 (2024): 1802–1824, 10.1002/ajh.27422?utm=. 38943315 PMC11404783

[24] B. Li , R. King , B. Chan , C. S. Rollo , and C. Florkowski , “An Updated Diagnostic Range for Serum Free Light Chain Kappa/Lambda Ratio Using Freelite Reagents on BN II or Optilite,” Pathology 56 (2024): 732–734, 10.1016/j.pathol.2024.02.006. 38705800

[25] R. A. Kyle , M. A. Gertz , T. E. Witzig , et al., “Review of 1027 Patients With Newly Diagnosed Multiple Myeloma,” Mayo Clinic Proceedings 78 (2003): 21–33, 10.4065/78.1.21. 12528874

[26] S. Stifter , E. Babarovic , T. Valkovic , et al., “Combined Evaluation of Bone Marrow Aspirate and Biopsy Is Superior in the Prognosis of Multiple Myeloma,” Diagnostic Pathology 5 (2010): 30, 10.1186/1746-1596-5-30. 20482792 PMC2883968

[27] D. J. Bergstrom , R. Kotb , M. L. Louzada , H. J. Sutherland , S. Tavoularis , and C. P. Venner , “Consensus Guidelines on the Diagnosis of Multiple Myeloma and Related Disorders: Recommendations of the Myeloma Canada Research Network Consensus Guideline Consortium,” Clinical Lymphoma, Myeloma & Leukemia 20 (2020): e352–e367, 10.1016/j.clml.2020.01.017. 32249195

[28] S. V. Rajkumar , M. A. Dimopoulos , A. Palumbo , et al., “International Myeloma Working Group Updated Criteria for the Diagnosis of Multiple Myeloma,” Lancet Oncology 15 (2014): E538–E548, 10.1016/S1470-2045(14)70442-5. 25439696

